# Marfan syndrome: a case report and pictorial essay

**DOI:** 10.11604/pamj.2018.30.171.14363

**Published:** 2018-06-25

**Authors:** Poobalan Naidoo, Naresh Ranjith, Zuzile Zikalala, Scott Mahoney, Kevin Ho

**Affiliations:** 1 RK Khan Regional Hospital, Department of Internal Medicine and University of Kwa-Zulu Natal, South Africa; 2 RK Khan Regional Hospital, Department of Radiology and University of Kwa-Zulu Natal, South Africa; 3 King Edward VIII Tertiary Hospital, Department of Internal Medicine, South Africa; 4 Steve Biko Academic Hospital, Department of Cardiology and University of Pretoria, South Africa

**Keywords:** Connective tissue disease, marfan syndrome, radiological and vascular anomalies

## Abstract

We report a case of Marfan syndrome (MFS) in a South African patient, which is extraordinary because of the large constellation of clinical, radiological and vascular anomalies in a single patient. A literature search from 1950 to date did not show a similar report of such extensive clinical characteristics of MFS.

## Introduction

Marfan syndrome (MFS) is one of the most common inherited disorders of connective tissue [1, 2]. It is an autosomal dominant condition with a reported incidence of 1 in 3000 to 5000 individuals. There is a wide range of clinical severity associated with MFS with classic ocular, cardiovascular and musculoskeletal abnormalities, while some patients demonstrate significant involvement of the lung, skin and central nervous system. The diagnosis of MFS relies essentially on the fulfillment of clinical diagnostic criteria as outlined by the revised Ghent score [3]. We report a case of MFS which is extraordinary because of the large number of clinical, radiological and vascular anomalies occurring in a single subject. A search was conducted on MFS in humans from 1950 to date and showed close to 2000 citations, none of which presented with such extensive clinical characteristics of MFS in an individual. Furthermore, a Google^®^ and Yahoo^®^ search of images related to MFS did not show a similar case.

## Patient and observation

**Figure 1:**
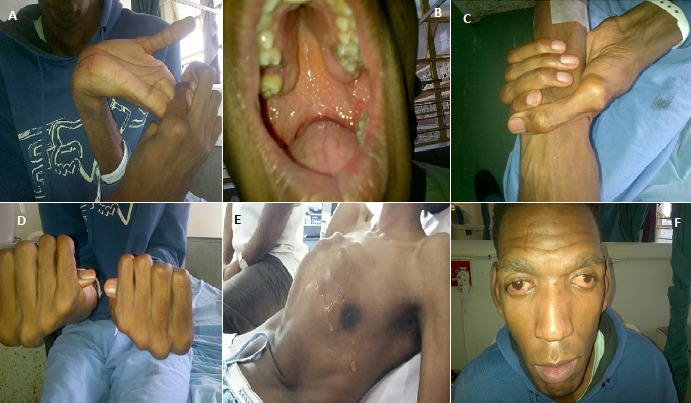
(A) wrist hypermobility; (B) high arched palate; (C) positive wrist sign; (D) positive thumb sign; (E) pectus carinatum; (F) dysmorphic facial features

An 18 year old black male, with no previous medical history, presented to the casualty department at the RK Khan Hospital in Durban, South Africa with atypical chest pain. The chest pain was present for a duration of 1 month and occurred in the early hours of the morning, lasting for approximately 3 minutes. There were no associated palpitations, autonomic symptoms, pre-syncope or syncope and the pain was not related to respiration or food intake. On further enquiry, the patient volunteered that his "different appearance" prompted him to search the internet to determine if other people had features similar to him. The internet search suggested that he may have MFS, and this was the primary reason for him to visit the hospital to find out if he indeed did have MFS. The patient did not have a family history of a similar problem. On general examination the patient was comfortable at rest, extremely tall (2.1 meters) and thin with typical musculoskeletal features of MFS. These included arachnodactyly with positive wrist and thumb signs, pectus carinatum deformity, hind foot valgus, dolichostenomelia (< 0.78), increased arm span/height, scoliosis, dolichochepaly, enophthalmos, downslanting palpebral fissures, malar hypoplasia, and retrognathia. His total systemic score was 10 based on the revised Ghent nosology. Figure 1 demonstrates the patients typical clinical signs of MFS. The patient was normotensive with a sinus bradycardia of 60 beats per minute. The first heart sound was soft with a murmur of mitral regurgitation graded as 4/5. There were no clinical features of infective endocarditis or cardiac failure. Respiratory examination demonstrated pectus carinatum and was otherwise normal. Ophthalmology examination conducted by an ophthalmologist revealed a left dense cataract with lens subluxation but no dislocation, iridodonesis, an old retinal detachment and minimal light perception. The right eye had a clear lens with no subluxation or dislocation, but showed iridodonesis with myopia. Laboratory tests were within normal limits (full blood count, urea and electrolytes, calcium, magnesium and phosphate, liver function tests, growth hormone, prolactin, troponin T, creatine kinase).

**Figure 2:**
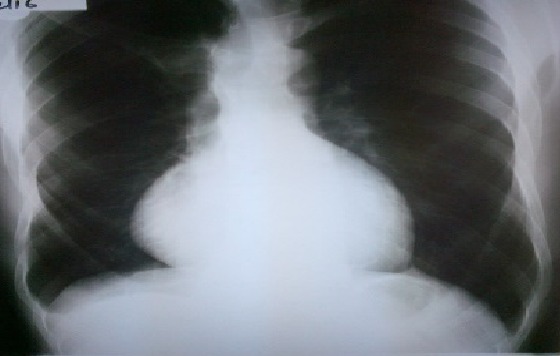
Chest radiograph of index case

**Figure 3:**
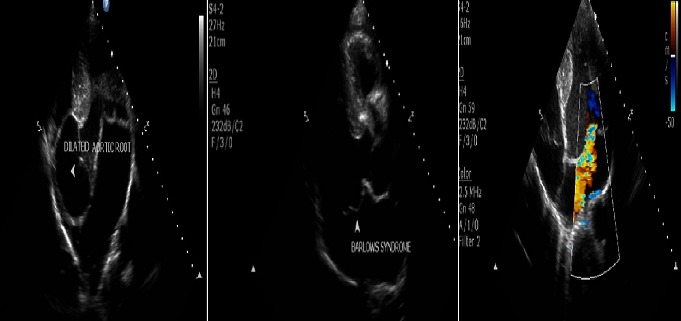
(A) echocardiographic findings of a dilated aortic root; (B) prolapsing mitral valve leaflet; (C) mitral valve regurgitation

**Figure 4:**
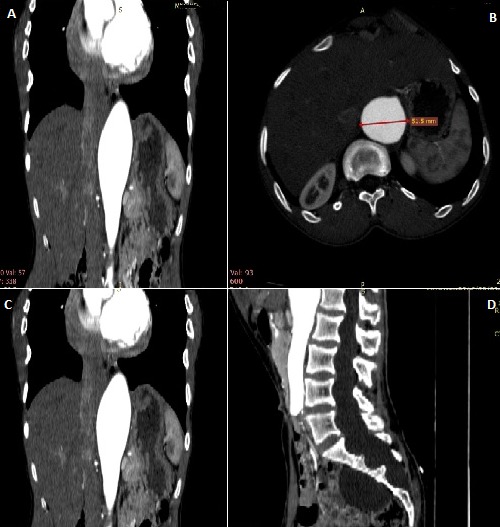
(A) transverse section showing abdominal aorta dilation; (B) dilation of abdominal aorta; (C) dilated aortic root; (D) lumbar-sacral spine deformity with dural ectasia; (E) sacral spine deformity with dural ectasia

Chest X-ray (Figure 2) showed an increased cardiothoracic ratio of greater the 50%. Echocardiography demonstrated prolapse of the anterior mitral valve leaflet with moderately severe mitral regurgitation and aortic root dilatation measuring 5 cm. The ejection fraction was 65% and no evidence of aortic dissection was noted (Figure 3). Computerised tomography (CT) (Figure 4 and its subsets) showed dilatation of the aortic sinuses of valsava extending to the ascending aorta. The aortic root was dilated measuring 6.3 X 6.4cm. The ascending aorta measured 3.1cm. There was no dissection or calcification of the aortic wall. The arch of the aorta and its main branches were within normal limits. The root of the main pulmonary artery was dilated measuring 4.1cm. The rest of the pulmonary arterial system was within normal limits. A suprarenal abdominal aortic aneurysm was noted with length 15.1 cm and diameter of 5 cm. There were no features of dissection, mural thrombi, or signs of imminent rupture. The right kidney had dual blood supply, with one artery arising from the inferior portion of the aneurysm and the other from the normal aorta inferiorly. The single left renal artery arose from the inferior aspect of the aneurysm. All renal arteries were patent and there was appropriate and symmetrical enhancement of the kidneys. The coeliac axis and the superior mesenteric arteries arose from the abdominal aortic aneurysm. The inferior mesenteric artery arose from the normal abdominal aorta. The lung fields were clear and the cranio-cervical junction was normal with no subluxation. There was pectus carinatum and a left scoliosis of the upper thoracic spine. Dural ectasia with posterior scalloping of the vertebral bodies was noted in the lumbar sacral spine. There was expansion of the sacral spinal canal and enlargement of the sacral foramen. The patient was counseled on his medical condition and offered surgery for his mitral valve and aortic root dilation. After consultation with his family he declined surgical intervention. He was discharged on losartan and subsequently lost to follow. Prior to discharge informed consent was obtained from the patient for publication of this case.

## Discussion

Marfan syndrome is almost exclusively inherited in an autosomal dominant manner with most patients harboring mutations involving the gene (FBN1) encoding the connective tissue protein fibrillin-1 [4]. In less than 10% with typical marfan phenotype, no mutations in FBN1 is identified and mutations in a gene encoding for transforming growth factor- beta receptors (TGFBR) maybe responsible [5]. While most individuals with MFS have an affected parent, about 25 % have MFS as a result of a de novo mutation. Our patient had no family history of MFS and genetic testing was not performed. It is important to note that even in the presence of a FBN1 mutation, the diagnosis of MFS relies on fulfillment of clinical diagnostic criteria according to the revised Ghent nosology. This patient had both cardinal features of the disease such as aortic root dilatation and ectopia lentis and systemic score of 10. Mortality is related to cardiac complications, which is frequently associated with aortic dissection. Although the index patient did not have evidence of dissection, the aortic root was markedly dilated. In addition, mitral valve prolapse (MVP) was identified on echocardiography, and this is found in about 40-54% of patients with MFS [6]. About 25% of patients with MFS and MVP have progressive disease with moderate to severe mitral regurgitation [6]. Heart failure attributable to MVP and regurgitation also represents a major source of morbidity and mortality and will require mitral valve replacement, similar to our patient. Based on current guidelines from the American Cardiac Society [7] this patient requires elective replacement of the aortic root and mitral valve replacement, but he declined surgical intervention. He was instructed to restrict strenuous activity and was commenced on losartan. Because of his sinus bradycardia, the patient was not started on beta blocker therapy, although this has been shown to decrease myocardial contractility and may also improve the elastic properties of the aorta [8]. While we respect the patient's decision not to undergo surgical intervention, the life span of patients with classic MFS is markedly decreased if untreated.

## Conclusion

Marfan syndrome is one of the most common inherited disorders of connective tissue and this index case merits consideration because of the wide variety of ocular, cardiovascular, musculoskeletal and vascular abnormalities occurring in a single patient.

## Competing interests

The authors declare no competing interests.
